# Identification and Characterisation of *Aedes aegypti* Aldehyde Dehydrogenases Involved in Pyrethroid Metabolism

**DOI:** 10.1371/journal.pone.0102746

**Published:** 2014-07-21

**Authors:** Nongkran Lumjuan, Jureeporn Wicheer, Posri Leelapat, Wej Choochote, Pradya Somboon

**Affiliations:** 1 Research Institute for Health Sciences, Chiang Mai University, Chiang Mai, Thailand; 2 Department of Parasitology, Faculty of Medicine, Chiang Mai University, Chiang Mai, Thailand; Institut national de la santé et de la recherche médicale - Institut Cochin, France

## Abstract

**Background:**

Pyrethroid insecticides, especially permethrin and deltamethrin, have been used extensively worldwide for mosquito control. However, insecticide resistance can spread through a population very rapidly under strong selection pressure from insecticide use. The upregulation of aldehyde dehydrogenase (*ALDH*) has been reported upon pyrethroid treatment. In *Aedes aegypti*, the increase in ALDH activity against the hydrolytic product of pyrethroid has been observed in DDT/permethrin-resistant strains. The objective of this study was to identify the role of individual ALDHs involved in pyrethroid metabolism.

**Methodology/Principal Findings:**

Three ALDHs were identified; two of these, ALDH9948 and ALDH14080, were upregulated in terms of both mRNA and protein levels in a DDT/pyrethroid-resistant strain of *Ae. aegypti*. Recombinant ALDH9948 and ALDH14080 exhibited oxidase activities to catalyse the oxidation of a permethrin intermediate, phenoxybenzyl aldehyde (PBald), to phenoxybenzoic acid (PBacid).

**Conclusions/Significance:**

ALDHs hav*e* been identified in association with permethrin resistance in *Ae. aegypti*. Characterisation of recombinant ALDHs confirmed the role of this protein in pyrethroid metabolism. Understanding the biochemical and molecular mechanisms of pyrethroid resistance provides information for improving vector control strategies.

## Introduction

Pyrethroids, synthetic insecticides analogous to natural pyrethrin, have been widely used throughout the world for the control of insects. Pyrethroids are divided into two groups based on their chemical structures. Type I pyrethroids, such as permethrin, lack an α-cyano group, whereas type II pyrethroids, such as deltamethrin and cypermethrin, contain an α-cyano group. However, the extensive use of these insecticides has led to insecticide resistance in insect populations [1], [2], [3]. Resistance to pyrethroids can be divided into two main mechanisms: an alteration in the target site of the insecticide or increased expression of metabolic detoxification enzymes. Pyrethroids act by targeting sodium channels, leading to neurotoxic effects [4]. Several point mutations in the voltage-gated sodium channel gene are associated with DDT and pyrethroid resistance [5], [6], [7], [8], [9]. In metabolic resistance, enhanced activity of enzymes in metabolic pathways in insects leads to insecticides being detoxified or sequestered before they reach the target site. Overexpression of detoxification enzymes such as cytochromes P450 (CYPs), glutathione S-transferases (GSTs) and carboxylesterases (CEs) have been well documented in pyrethroid resistance in insects [10], [11], [12].

Pyrethroids are mainly metabolised through the hydrolysis of the ester linkage followed by the oxidation of their component alcohol and acid moieties [13]. Pyrethroids have been extensively studied in humans and rats, indicating that both types are mainly hydrolysed by CEs to produce 3-phenoxybenzyl alcohol (PBalc) [14], [15], whereas they are mainly oxidised by P450s, alcohol dehydrogenases (ADHs) and aldehyde dehydrogenases (ALDHs) [16], [17]. ALDHs have been investigated as enzymes that are important in the oxidation of permethrin in mammals for their oxidation of intermediate products of pyrethroid to carboxylic acid [18]. In the mosquito *Anopheles gambiae*, the up-regulation of ALDH after exposure to permethrin has been reported [19]. Enzyme-based metabolite assays also indicated that the catalytic activity of P450s, ADHs and ALDHs were increased in microsomal fractions of a DDT/permethrin-resistant strain (PMD-R) of *Aedes aegypti* from Thailand [20]. In our preliminary study using a proteomic approach, crude homogenates of 4^th^ instar larvae of *Aedes* mosquitoes were partially purified using glutathione agarose columns. Bound fractions were collected, concentrated and separated by 2-dimensional gel electrophoresis. The result indicated that a detoxification enzyme, ALDH (AAEL014080 in VectorBase), was upregulated in the PMD-R strain relative to the laboratory susceptible strain (unpublished data). However, the ability of individual ALDHs isoforms to metabolise permethrin in mosquito has not yet been investigated.

The present study aimed to identify the ALDH genes responsible for permethrin resistance in *Ae. aegypti*. The individual ALDHs that are involved in permethrin resistance were characterised, and their expression patterns were analysed. Recombinant proteins were produced, and the in vitro metabolism of permethrin and its hydrolysis products were determined.

## Materials and Methods

### Materials

Cis/Trans-permethrin was purchased from Chem Service (West Chester, PA). Permethrin metabolites, 3-phenoxybenzyl alcohol (PBalc, 98% purity), 3-phenoxybenzylaldehyde (PBald, 98% purity) and 3-phenoxybenzoic acid (PBacid, 98% purity) including β-Nicotinamide adenine dinucleotide (NAD^+^) were purchased from Sigma (St. Louis, MO).

### Mosquito strains

The PMD and PMD-R strains originated from Chiang Mai Province, Thailand [21]. The PMD strain was resistant to DDT, whereas the PMD-R strain was resistant to both DDT and permethrin. The New Orleans strain was an insecticide-susceptible laboratory strain of *Ae. aegypti*.

### Database search and sequence alignment

A preliminary study using 2-dimensional gel electrophoresis demonstrated that expression of ALDH (AAEL014080) was increased in the PMD-R strain relative to the NO and PMD strains at the larval stage (unpublished data). The protein sequence of a known ALDH (AAEL014080) was used as a query for a BLAST search of the *Aedes aegypti* sequences in VectorBase. Deduced amino acid sequences of ALDHs were aligned using ClustalW [22].

### Identification of *ALDH* genes

The oligonucleotide primers were designed based on the sequences of ALDH in VectorBase (Table S1). The full-length cDNAs of ALDH genes from *Ae. aegypti* were amplified using Taq DNA polymerase (Qiagen) as described by the manufacturer's protocol. PCR parameters consisted of 35 cycles of 30 s at 95°C, 30 s at 55°C, and 1.5 min at 72°C. PCR products were cloned into the pGEM-T easy Vector (Promega) and then transformed into JM109 competent *Escherichia coli* cells. The plasmid DNA was submitted to 1^st^ BASE Laboratories (Malaysia) for sequencing to verify the integrity of genes.

### Quantitative PCR analysis

Total RNA was extracted from 3 biological replicate sets (10 mosquitoes per replicate) of 4^th^ instar larvae, pupae, and one-day-old adult males or females from each of the three strains using the TRIzol plus RNA Purification System (Invitrogen). Complementary DNA was synthesised using SuperScript III reverse transcriptase (Gibco) as described in the manufacturer's protocol. Quantitative PCR was performed as previously described, using QuantiFast SYBR Kit's protocol (Qiagen) [23]. The primers used are shown in Table S2. The PCR parameters consisted of 2 steps of 95°C for 5 min and 35 cycles of 95°C for 10 s, 60°C for 35 s, followed by a dissociation step.

### Construction of plasmids and expression of ALDHs

Total RNA was extracted from whole mosquitoes of the PMD-R strain using Trizol reagent (Sigma). Complementary DNA was synthesised using SuperScript III reverse transcriptase (Gibco) as described in the manufacturer's protocol. PCR products generated with ProofStart DNA polymerase (Qiagen) using gene specific primers (Table S1) were cloned into the pET 100-D/TOPO vector using the Champion pET directional TOPO Expression kit according to the manufacturer's instruction (Invitrogen). The construct was verified by DNA sequencing. The plasmids containing the ALDH genes were transformed into *E. coli* BL21 Star (DE3). The recombinant proteins were produced after induction with isopropyl β−D-thiogalactoside at 37°C or room temperature for 4 h.

### Protein purification

The pET 100-D/TOPO vector encodes an N-terminal polyhistidine (6xHis) fused to the recombinant protein. Protein purification was performed using HisTrap Ni affinity column (GE Healthcare) as described previously [23]. The protein purity was verified by 12.5% polyacrylamide gel electrophoresis and Coomassie staining. The protein concentration was determined by the Bradford method using the Bio-Rad protein-assay dye reagent and bovine serum albumin as a standard [24].

### Western Blot analysis

Western blot analysis was performed as previously described [21]. The membrane was probed with 1∶50,000 and 1∶100,000 dilutions of polyclonal antibodies against ALDH9948 and ALDH14080, respectively. The bound antiserum was detected by incubation with a 1∶50,000 dilution of Peroxidase-labelled Anti-Rabbit Antiserum followed by visualisation using ECL Advanced Blotting Detection Kit (Amersham Bioscience).

### Enzyme activity

ALDH activity against PBald was measured as described previously [16]. Briefly, the substrate mixture contained 1 mM EDTA, 0.1 mM pyrazole and 2.5 mM NAD(P)^+^ in 33 mM Phosphate buffer, pH 8.2. The enzyme was incubated with the substrate mixture at 37°C, and the reaction rate was determined by the formation of NAD(P)H at 340 nm in 4 min. The esterase assay was conducted as described previously, by measuring the hydrolysis of p-nitrophenyl acetate (pNPA) to the products p-nitrophenol (pNP) and acetate [25]. Kinetic studies were performed by varying the concentration of PBald in the presence of NAD^+^. The results were analysed by non-linear regression analysis using GraphPad Prism 4 software.

### PBald oxidation by recombinant ALDHs

ALDH activity was measured by the oxidation of PBald to PBacid, as detected by HPLC. The assay was modified from the method described previously [17]. Briefly, 20 µg of recombinant ALDHs were incubated with 0.4 mM PBald in the presence of 3 mM NAD^+^ in 0.1 M Tris-Cl buffer, pH 7.4 at 37°C for 10 min. Pyrene was then added as an internal control. The reaction mixture was extracted with 1.5 ml of chloroform. This procedure was repeated in triplicate. The chloroform extracts were then pooled, air-dried and analysed with HPLC.

HPLC was performed with a Shimadzu LC 20-A Series (Shimadzu) using a Nova-Pak C18 column (3.9×150 mm; Waters). The extract was resuspended in 200 µl of acetonitrile. The mixture (10 µl) was injected into the column at a flow rate of 1 ml/min. The gradient elution was performed at 35°C, and the detection wavelength was 230 nm. Peaks were integrated into peak area with the LC Solution (Shimadzu). ALDH activity was calculated as the formation of PBacid/min/mg protein. The concentration of PBacid was determined by comparison with a known concentration of PBacid.

## Results

### Identification of *Ae. aegypti* ALDHs

**Figure 1:**
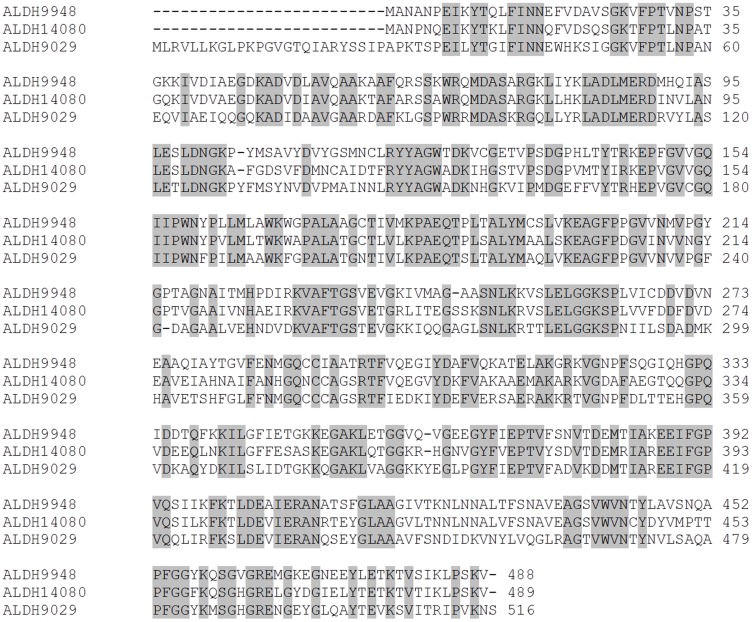
Deduced amino acid sequences of *Ae. aegypti* ALDH9948 and ALDH14080. Sequences shown are from the PMD-R strain. The amino acid sequences were aligned using ClustalW. Letters in bold indicate 100% conservation between the 3 sequences. Dashes are used to denote gaps introduced for maximum alignment.

The DNA sequence of ALDH (AAEL014080) in *Ae. aegypti* was retrieved from VectorBase (http://www.vectorbase.org), and it is located in supercontig 1.1002. Close paralogues of ALDH (AAEL014080), ALDH (AAEL009948) and ALDH (AAEL009029) were included in the experiment to expand for genes of interest that were found on supercontigs 1.440 and 1.363, respectively (Figure S1). The deduced amino acid sequences of these three ALDHs are shown in Figure 1.

### Quantitative PCR analysis

**Figure 2:**
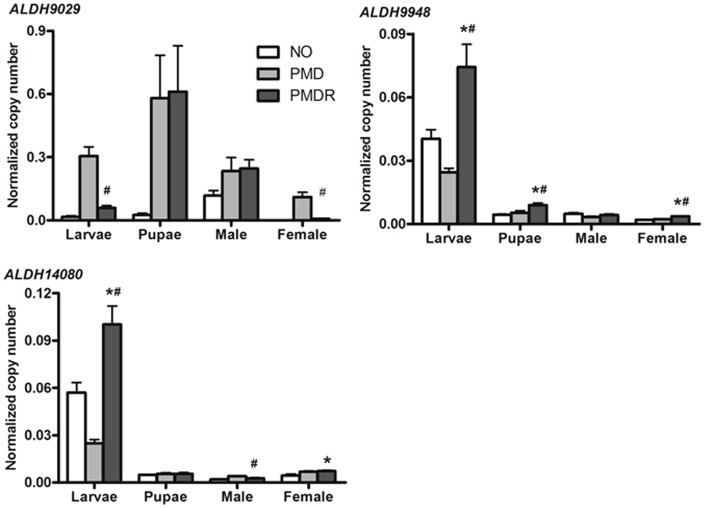
Transcription profiles of *ALDH9029*, *ALDH9948* and *ALDH14080* in three strains of *Ae. aegypti*. Complementary DNA from three different biological replicates (ten mosquitoes each) was used as templates. Four life-stages were analysed: larvae (L), pupae (P), adult male (M), and adult female (F). Each sample was analysed in duplicate in each experiment, and the results were averaged from three independent experiments. The mRNA copy numbers were determined by comparison with known concentrations of standard plasmids and normalised against the copy number of the ribosomal S7 transcript. Error bars indicate standard error of the mean. Statistically significant differences were evaluated with ANOVA followed by Tukey's multiple comparison test (*p<0.05 versus New Orleans strain; ^#^ *p*<0.05 versus PMD strain).

To determine whether the ALDH genes were overexpressed at the transcriptional level, real-time PCR was performed in three *Ae. aegypti* strains, the NO susceptible strain and the PMD and PMD-R strains, at three developmental stages. *ALDH14080* was significantly upregulated in the larvae and females of the PMD-R strain relative to the NO strain (Figure 2). *ALDH9948* mRNA levels were significantly upregulated in all life stages except the adult male (*p*<0.001 in larva and adult female, and *p*<0.05 in pupa) when compared to the PMD strain. *ALDH14080* expression was significantly higher in the larval stage of PMD-R only relative to PMD. In contrast, there is no evidence of upregulation of *ALDH9029* mRNA in the PMD-R strain when compared to the PMD strain in any life stage (). These results show that upregulation of *ALDH9948* and *ALDH1408*0 may confer resistance to permethrin.

### Western Blot analysis

**Figure 3:**
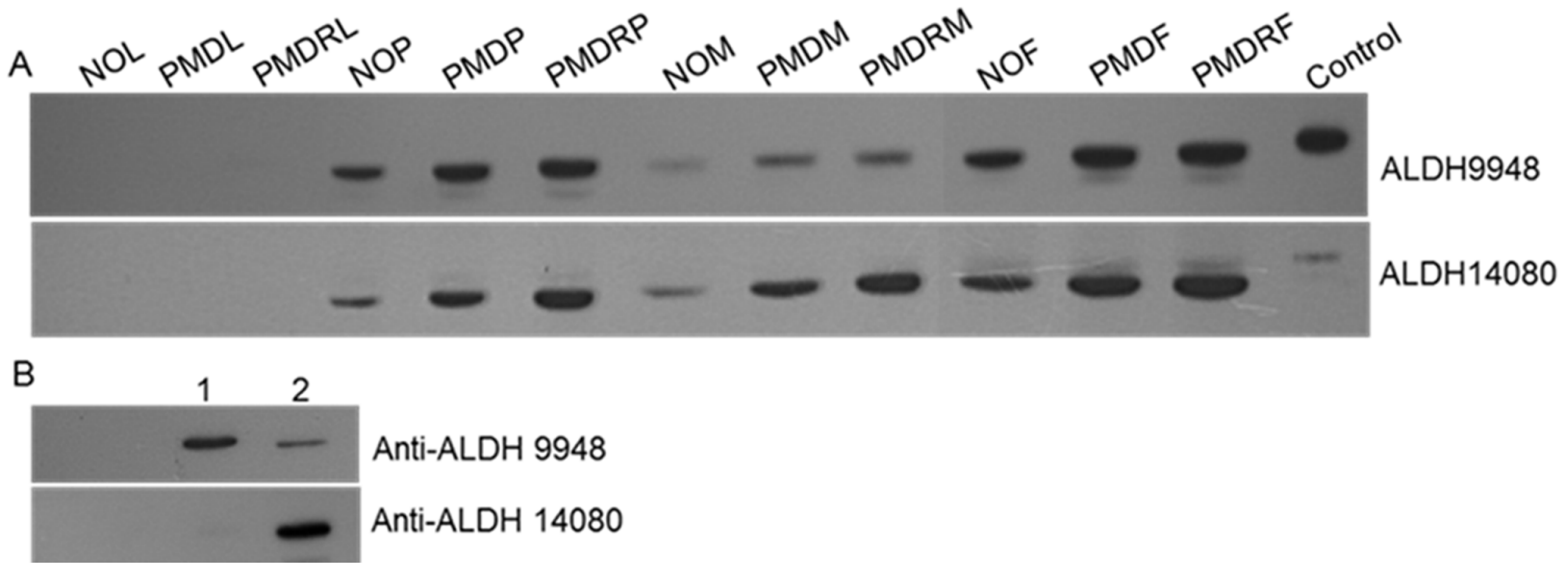
Western blot analysis of ALDH9948 and ALDH14080. (A) Elevated protein of ALDH9948 and ALDH14080 in PMD-R strain. Fifty micrograms of protein from New Orleans (NO), PMD and PMD-R strains in four life stages; larva (L), pupae (P), adult male (M) and adult female (F) including purified recombinant His-tagged ALDH9948 and 14080 (25 ng each) were resolved by SDS-PAGE. Proteins were transferred to a nitrocellulose membrane and probed with anti-ALDH9948 and anti-ALDH14080. Peroxidase labelled anti-rabbit antibody was used as a secondary antibody. Proteins were visualised by enhancing the chemiluminescence using ECL Advanced Blotting Detection Kit (Amersham Biosciences). Equal protein loading was confirmed by staining the membrane with Ponceus S. (B) Determination of antibodies specificity by western blot. Fifty nanograms of non-fusion ALDH9948 and ALDH14080 (Lane1, 2 and 3, respectively) were resolved in SDS-PAGE. Western blotting was performed as described.

To confirm the expression of ALDHs at the protein level, western blots were performed using specific polyclonal antibodies against ALDH9948 and ALDH14080. To validate the specificity of these polyclonal antibodies, immuno-cross-reactivity between ALDH9948 and ALDH14080 was investigated. The polyclonal antibody for ALDH9948 exhibited low-level cross-reactivity with ALDH14080 (Figure 3B), whereas the anti-ALDH14080 antibody was observed to have high specificity. Protein expression profiles of ALDH were investigated in crude homogenates of four developmental stages of three *Ae. aegypti* strains. Expression levels of ALDH9948 and ALDH14080 were increased in the PMD-R strain in almost all developmental stages (pupae and adult males and females), except for larvae when compared to the NO and PMD strains (Figure 3A). In all three strains, no visible bands of ALDHs were detected in the larval stage, whereas strong bands were presented in pupae and adult males and females. Meanwhile, crude homogenates from the larval stage gave no smearing bands when stained with Coomassie blue, indicating no protein degradation. The expression of rat ALDH has been reported to increase with age [26]. This might indicate that early stages express ALDH proteins at low levels that could not be detected in the small number of larvae used in this study.

### Recombinant protein expression

To determine whether the *Ae. aegypti* ALDHs contribute to permethrin metabolism, recombinant ALDHs were produced, and the ability of these proteins to metabolise permethrin was determined. The full-length sequences of two ALDHs, ALDH9948 and ALDH14080, were amplified by PCR using cDNA templates from the PMD-R strain and subcloned into the *E. coli* expression vector pET 100-D/TOPO. Expression of His_6_-tagged ALDHs in *E. coli* BL21 Star (DE3) yielded soluble recombinant proteins at the 37°C expression temperature. The purity of His_6_-tagged recombinant ALDHs was verified in 12.5% SDS-PAGE and corresponded to the predicted size of approximately 65 kDa (Figure S2).

### Biochemical characterisation of ALDHs

Both His_6_-tagged recombinant ALDHs possess ALDH activity to catalyse the oxidation of intermediate aldehyde of permethrin, PBald. The ALDH activity was measured by spectrophotometry, mediated by the formation of NAD(P)H as products of the reaction. The oxidation reactions of recombinant ALDH9948 and ALDH14080 required either NAD^+^ or NADP^+^ as a cofactor; however, these enzymes prefer NAD^+^ to NADP^+^ (Table 1). It has been noted that most ALDHs prefer to use NAD^+^ over NADP^+^ as a cofactor [27]. Generally, ALDHs exhibit esterase activity in vitro [28]. In this study, recombinant ALDHs also have esterase activities that catalyse the hydrolysis of p-nitrophenyl acetate to produce p-nitrophenol and acetate (Table 1). The highest esterase activity belongs to recombinant ALDH14080, with a specific activity of 13.11±0.98 µmole/min/mg proteins.

**Table 1:** Substrate specificity of *Ae. aegypti* recombinant ALDH isoforms.

	Substrate/cofactor	ALDH 9948	ALDH 14080
Esterase activity (µmole/min/mg)	pNPA	13.11±0.98	0.14±0.02
ALDH activity	PBald/NAD^+^	483±9	254±24
(nmole/min/mg)	PBald/NADP^+^	58±4	19±2

ALDH activity was performed in the presence 4 mM PBald and 2.5 mM NAD(P)^+^. The oxidation of PBald was monitored by the formation of NAD(P)H.

Kinetic parameters of purified ALDHs were determined using PBald and NAD^+^ as substrate and cofactor, respectively. Michaelis-Menten constants (*K*_m_) for ALDH9948 and ALDH14080 were 153.8±30.0 and 34.4±6.8 nM, respectively, in respect to PBald (Table 2). The (*V*_max_/*K*_m PBald_) value of ALDH14080 was higher than that of ALDH9948, indicating the catalytic efficiency of this enzyme at low substrate concentrations.

**Table 2:** Kinetic parameter of *Ae. aegypti* recombinant ALDH isoforms.

Enzyme	*V*_max_ (nmole NADH/min/mg)	PBald		NAD^+^	
		*K*_m_ (nM)	*V*_max_/*K*_m_	*K*_m_ (nM)	*V*_max_/*K*_m_
ALDH 9948	627.4±34.0	153.8±30.8	4.1	139.1±27.9	3.2
ALDH 14080	208.2±9.8	34.4±6.8	6.1	193.8±34.5	1.3

Kinetic studies were performed by varying the concentration of PBald and cofactor NAD^+^ at fixed saturated concentrations of NAD^+^ and PBald, respectively. The oxidation of PBald to PBacid was monitored by the formation of NADH in the reaction at 37°C for 4 min. Three independent assays were performed. The results are shown as the mean ± SE.

To determine whether recombinant ALDHs readily oxidised PBald, HPLC was performed to identify the product of PBacid. The metabolite profile of *trans*/*cis*-permethrin is shown in Figure S3. Pyrene was spiked as an internal control, given the extraction recovery range of 81–97%. The HPLC results indicated that PBald was oxidised by recombinant ALDH9948 and ALDH14080 with specificities of 1192±55 and 1119±14 nmole PBacid formed/min/mg protein, respectively (Table 3). Because recombinant ALDHs exhibit esterase activity, the ability of these enzymes to catalyse the hydrolysis of the parent permethrin was investigated. The incubation of recombinant ALDHs with *trans*/*cis*-permethrin did not produce PBalc, suggesting that ALDHs are not associated with permethrin hydrolysis (data not shown). The incubation of denatured recombinant ALDHs with PBald in the presence of NAD^+^ did not produce PBacid, indicating that the oxidation of PBald was mediated by recombinant ALDHs.

**Table 3:** Specific activity of *Ae.aegypti* recombinant ALDH isoforms to oxidise PBald.

Enzyme	Specific activity (nmole PBacid formed/min/mg protein)
ALDH 9948	1192±55
ALDH 14080	1119±14

Recombinant ALDH (5 µg) was incubated with 2 mM PBald in the presence of 3 mM NAD+ in 0.1 M Tris-Cl buffer, pH 7.4 at 37°C for 10 min. PBacid formation was determined by HPLC as described. Three independent assays were performed. The results are shown as the mean ± SE.

## Discussion

Overexpression of detoxification genes has been well documented in association with insecticide resistance of many insect species. P450s, GSTs and CEs are primarily implicated in the detoxification of insecticides in insects. It has been reported that P450s contribute to resistance in all classes of insecticides [29]. The upregulation of several P450s, particularly those belonging to the CYP6Z, CYP6M or CYP9J subfamilies, has been reported to be involved in resistance to pyrethroids in mosquitoes [30], [31], [32], [33]. Some species, including *Ae. aegypti* CYP9J32, *An. gambiae* CYP6M2 and *An. gambiae* CYP6Z8, have the ability to metabolise pyrethroids [34], [35], [36]. GSTs, especially GSTE2, GSTE4 and GSTE7, were also observed to be overexpressed in resistant populations [32], [33], [37]. Recombinant GSTE2-2 showed DDT dehydrochlorinase activity to metabolise DDT, but the recombinant GSTE7-7 did not appear to metabolise DDT. Therefore, the role of GSTE7 in insecticide resistance remains unclear [21]. A recent study suggested that a single point mutation of *GSTe2* (L119F) associated with metabolic resistance to DDT and permethrin in mosquito *An. funetus* [38]. Many genes encoding CE enzymes were identified to be upregulated in organophosphate-, carbamate- and pyrethroid-resistant insects [39].

However, other genes that are responsible for insecticide resistance cannot be excluded. To date, microarray technology has been utilised to expand the number of detoxification genes and has identified new relevant genes that might be involved in metabolic resistance [19], [32], [33], [40], [41], [42]. Aside from P450s, GSTs and CEs, microarray data also identified secondary detoxification genes that may confer insecticide resistance. For example, aldo-ketoreductase, an NAD(P)(H) oxidoreductases that catalyse the reduction of aldehydes to alcohols, was over-transcribed in temephos- and permethrin- selected strain of *Ae. aegypti* [43], [44]. UDP-glucuronosyltransferases (UGTs), phase II detoxification enzymes involved in the conjugation of xenobiotics, were also identified as upregulated after permethrin exposure and in response to carbamate, respectively. ALDHs were also found to be upregulated in insecticide resistance in insects [19], [40]. However, the functions of these enzymes in insecticide detoxification require further investigation. In mammals, the oxidation of pyrethroids was catalysed by ALDH [16]. A study in insecticide metabolism revealed the important role of ALDH in the detoxification of pyrethroid in mosquito [20]. Multiple detoxification enzymes were identified as a target of pyrethroid activity-based probes in rat proteome, including P450s, UDP-glucuronosyltransferases, Flavin-containing monooxygenase and ALDH [45].

Aldehyde dehydrogenases are a family of enzymes that oxidise a broad range of endogenous, xenobiotic and lipid peroxidation products that contain the highly reactive aldehyde to their corresponding carboxylic acid [25]. In mammals, ALDHs are involved in both the detoxification of aldehydes and the biosynthesis of pheromones [27]. However, few studies of ALDHs have been reported in insects. In *Drosophila*, ALDHs play a vital role in ethanol metabolism by mediating the oxidation of acetaldehyde to acetate, which is involved in ethanol resistance [27], [28], [46], [47].

In this study, transcript levels for three of the *Ae. aegypti* ALDH genes were quantified. *ALDH9948* was significantly overexpressed in the insecticide-resistant PMD-R strain in almost all developmental stages, except adult males, when compared to the susceptible PMD line. In contrast, *ALDH14080* was upregulated relative to the PMD strain only in the larval stage. Quantitative PCR results revealed that insecticide selection increased the expression of these ALDHs, although the overexpression was not observed in all life stages. The altered expression of ALDH9948 and ALDH14080 was confirmed at the protein level, indicating that the increase in these proteins is strongly associated with resistance to permethrin. Inconsistencies between the mRNA and protein levels of the same gene may be caused by differences in post-translational regulation between the different developmental stages. Although high levels of ALDH mRNA were found in the larval stage, there was no protein detected by western blot, suggesting that the protein may be expressed at a level below the detection limit in early stages. However, low-abundance ALDH was detected by 2D-gel electrophoresis from a large sample of larvae used in combination with the sub-proteome approach for the enrichment of low-abundance proteins. The recombinant ALDH isoforms exhibited oxidase activity to catalyse the oxidation of aldehyde moiety of pyrethroids, but subcellular localisation of individual ALDHs was not investigated further in this study. These experiments suggested that ALDH9948 and ALDH14080 may play a role in insecticide resistance to permethrin in the PMD-R strain of *Ae. aegypti*.

Collectively, in *Ae. aegypti*, it has been reported that parental permethrin can be hydrolysed in vitro. Our previous study demonstrated that the formation of PBacid was decreased in the presence of an esterase inhibitor, BNPP, suggesting the function of esterases in permethrin metabolism [20]. The importance of particular CEs in pyrethroid detoxification has not yet been studied. However, it has been proposed that non-specific esterases may be involved in pyrethroid hydrolysis in insects [48]. A recent study demonstrated that both PBalc and PBald were oxidised by *Ae. aegypti* CYP6Z8 [34]. In addition, our finding also clearly revealed that recombinant ALDH9948 and ALDH14080 have the ability to catalyse the oxidation of PBald. The results of this study indicate the role of *Ae. aegypti* ALDHs in pyrethroid degradation pathway and this knowledge will improve our ability to manage insecticide resistance in the field such as the use of synergists to increase the efficacy of certain insecticides.

In conclusion, we identified two ALDHs that are upregulated in permethrin-resistant *Ae. aegypti* mosquitoes in Thailand. Functional characterisation of recombinant ALDHs clearly demonstrates that these enzymes are capable of metabolising PBald. This report indicates the importance of *Ae. aegypti* ALDHs in permethrin degradation.

## Supporting Information

Figure S1 **Phylogenetic relationship of ALDHs in** ***Ae. aegypti*** **(AAEL) with** ***An. gambiae*** **ALDHs (AGAP).**
(DOCX)

Figure S2 **SDS-PAGE analysis of His-tagged recombinant ALDHs in** ***Ae. aegypti*** **produced in** ***E. coli*** **BL21 Star (DE3).**
(DOCX)

Figure S3 **Chromatograms of PBald oxidation to PBacid by recombinant aldehyde dehydrogenase.**
(DOCX)

Table S1 **Sequences of oligonucleotide primers used to amplify the cDNA full-length of ALDHs for in vitro protein expression.**
(DOCX)

Table S2 **Sequences of oligonucleotide primers used to amplify the fragment of** ***Ae. aegypti*** **ALDHs for quantitative PCR.**
(DOCX)

Table S3 **Quantitative PCR results of** ***Ae. aegypti*** **ALDH.**
(DOCX)

## References

[1] Chareonviriyaphap T, Bangs MJ, Suwonkerd W, Kongmee M, Corbel V, et al (2013) Review of insecticide resistance and behavioral avoidance of vectors of human diseases in Thailand. Parasit Vectors 6: 280. 24294938 10.1186/1756-3305-6-280 PMC3850650

[2] Hemingway J, Field L, Vontas J (2002) An overview of insecticide resistance. Science 298: 96–97. 12364782 10.1126/science.1078052

[3] Maharaj R (2011) Global trends in insecticide resistance and impact on disease vector control measures. Insect Physiol 3: 27–33.

[4] Soderlund DM, Clark JM, Sheets LP, Mullin LS, Piccirillo VJ, et al (2002) Mechanisms of pyrethroid neurotoxicity: implications for cumulative risk assessment. Toxicology 171: 3–59. 11812616 10.1016/s0300-483x(01)00569-8

[5] Brengues C, Hawkes NJ, Chandre F, McCarroll L, Duchon S, et al (2003) Pyrethroid and DDT cross-resistance in *Aedes aegypti* is correlated with novel mutations in the voltage-gated sodium channel gene. Med Vet Entomol 17: 87–94. 12680930 10.1046/j.1365-2915.2003.00412.x

[6] Martins AJ, Lima JB, Peixoto AA, Valle D (2009) Frequency of Val1016Ile mutation in the voltage-gated sodium channel gene of *Aedes aegypti* Brazilian populations. Trop Med Int Health 14: 1351–1355. 19735371 10.1111/j.1365-3156.2009.02378.x

[7] Rajatileka S, Black WCt, Saavedra-Rodriguez K, Trongtokit Y, Apiwathnasorn C, et al (2008) Development and application of a simple colorimetric assay reveals widespread distribution of sodium channel mutations in Thai populations of *Aedes aegypti* . Acta Trop 108: 54–57. 18801327 10.1016/j.actatropica.2008.08.004

[8] Saavedra-Rodriguez K, Urdaneta-Marquez L, Rajatileka S, Moulton M, Flores AE, et al (2007) A mutation in the voltage-gated sodium channel gene associated with pyrethroid resistance in Latin American *Aedes aegypti* . Insect Mol Biol 16: 785–798. 18093007 10.1111/j.1365-2583.2007.00774.x

[9] Yanola J, Somboon P, Walton C, Nachaiwieng W, Somwang P, et al (2011) High-throughput assays for detection of the F1534C mutation in the voltage-gated sodium channel gene in permethrin-resistant *Aedes aegypti* and the distribution of this mutation throughout Thailand. Trop Med Int Health 16: 501–509. 21342372 10.1111/j.1365-3156.2011.02725.x

[10] Feyereisen R (2005) Insect Cytochrome P450. In: Gilbert LI, Iatrou K, Gill SS, editors. Comprehensive Molecular Insect Science Elsevier pp. 1–77.

[11] Oakeshott JG, Devonshire AL, Claudianos C, Sutherland TD, Horne I, et al (2005) Comparing the organophosphorus and carbamate insecticide resistance mutations in cholin- and carboxyl-esterases. Chem Biol Interact 157–158: 269–275. 10.1016/j.cbi.2005.10.041 16289012

[12] Ranson H, Hemingway J (2005) Glutathione transferases In: Gilbert LI, Iatrou K, Gill SS, editors. Comprehensive Molecular Insect Science - Pharmacology: Elsevier. pp. 383–402.

[13] Kaneko H (2011) Pyrethroids: mammalian metabolism and toxicity. J Agric Food Chem 59: 2786–2791. 21133409 10.1021/jf102567z

[14] Nishi K, Huang H, Kamita SG, Kim IH, Morisseau C, et al (2006) Characterization of pyrethroid hydrolysis by the human liver carboxylesterases hCE-1 and hCE-2. Arch Biochem Biophys 445: 115–123. 16359636 10.1016/j.abb.2005.11.005 PMC1444892

[15] Ross MK, Borazjani A, Edwards CC, Potter PM (2006) Hydrolytic metabolism of pyrethroids by human and other mammalian carboxylesterases. Biochem Pharmacol 71: 657–669. 16387282 10.1016/j.bcp.2005.11.020

[16] Choi J, Rose RL, Hodgson E (2002) In vitro human metabolism of permethrin: the role of human alcohol and aldehyde dehydrogenase. Pest Biochem Physiol 22: 249–261.

[17] Nakamura Y, Sugihara K, Sone T, Isobe M, Ohta S, et al (2007) The in vitro metabolism of a pyrethroid insecticide, permethrin, and its hydrolysis products in rats. Toxicology 235: 176–184. 17451859 10.1016/j.tox.2007.03.016

[18] Hodgson E (2003) In vitro human phase I metabolism of xenobiotics I: pesticides and related compounds used in agriculture and public health. J Biochem Mol Toxicol 17: 201–206. 12898643 10.1002/jbt.10080

[19] Vontas J, Blass C, Koutsos AC, David JP, Kafatos FC, et al (2005) Gene expression in insecticide resistant and susceptible *Anopheles gambiae* strains constitutively or after insecticide exposure. Insect Mol Biol 14: 509–521. 16164607 10.1111/j.1365-2583.2005.00582.x

[20] Somwang P, Yanola J, Suwan W, Walton C, Lumjuan N, et al (2011) Enzymes-based resistant mechanism in pyrethroid resistant and susceptible *Aedes aegypti* strains from northern Thailand. Parasitol Res 109: 531–537. 21336645 10.1007/s00436-011-2280-0

[21] Lumjuan N, McCarroll L, Prapanthadara LA, Hemingway J, Ranson H (2005) Elevated activity of an Epsilon class glutathione transferase confers DDT resistance in the dengue vector, *Aedes aegypti* . Insect Biochem Mol Biol 35: 861–871. 15944082 10.1016/j.ibmb.2005.03.008

[22] Thompson JD, Higgins DG, Gibson TJ (1994) CLUSTAL W: improving the sensitivity of progressive multiple sequence alignment through sequence weighting, position-specific gap penalties and weight matrix choice. Nucleic Acids Res 22: 4673–4680. 7984417 10.1093/nar/22.22.4673 PMC308517

[23] Lumjuan N, Rajatileka S, Changsom D, Wicheer J, Leelapat P, et al (2011) The role of the *Aedes aegypti* Epsilon glutathione transferases in conferring resistance to DDT and pyrethroid insecticides. Insect Biochem Mol Biol 41: 203–209. 21195177 10.1016/j.ibmb.2010.12.005

[24] Bradford MM (1976) A rapid and sensitive method for the quantitation of microgram quantities of protein utilizing the principle of protein-dye binding. Anal Biochem 72: 248–254. 942051 10.1016/0003-2697(76)90527-3

[25] Marchitti SA, Brocker C, Stagos D, Vasiliou V (2008) Non-P450 aldehyde oxidizing enzymes: the aldehyde dehydrogenase superfamily. Expert Opin Drug Metab Toxicol 4: 697–720. 18611112 10.1517/17425250802102627 PMC2658643

[26] Yoon M, Madden MC, Barton HA (2006) Developmental expression of aldehyde dehydrogenase in rat: a comparison of liver and lung development. Toxicol Sci 89: 386–398. 16291827 10.1093/toxsci/kfj045

[27] Morse D, Meighen E (1984) Aldehyde Pheromones in Lepidoptera: Evidence for an Acetate Ester Precursor in *Choristoneura fumiferana* . Science 226: 1434–1436. 17789000 10.1126/science.226.4681.1434

[28] Fry JD, Bahnck CM, Mikucki M, Phadnis N, Slattery WC (2004) Dietary Ethanol Mediates Selection on Aldehyde Dehydrogenase Activity in *Drosophila melanogaster* . Integr Comp Biol 44: 275–283. 21676710 10.1093/icb/44.4.275

[29] Li X, Schuler MA, Berenbaum MR (2007) Molecular mechanisms of metabolic resistance to synthetic and natural xenobiotics. Annu Rev Entomol 52: 231–253. 16925478 10.1146/annurev.ento.51.110104.151104

[30] Bariami V, Jones CM, Poupardin R, Vontas J, Ranson H (2012) Gene amplification, ABC transporters and cytochrome P450s: unraveling the molecular basis of pyrethroid resistance in the dengue vector, *Aedes aegypti* . PLoS Negl Trop Dis 6: e1692. 22720108 10.1371/journal.pntd.0001692 PMC3373657

[31] Marcombe S, Mathieu RB, Pocquet N, Riaz MA, Poupardin R, et al (2012) Insecticide resistance in the dengue vector *Aedes aegypti* from Martinique: distribution, mechanisms and relations with environmental factors. PLoS One 7: e30989. 22363529 10.1371/journal.pone.0030989 PMC3283601

[32] Marcombe S, Poupardin R, Darriet F, Reynaud S, Bonnet J, et al (2009) Exploring the molecular basis of insecticide resistance in the dengue vector *Aedes aegypti*: a case study in Martinique Island (French West Indies). BMC Genomics 10: 494. 19857255 10.1186/1471-2164-10-494 PMC2770535

[33] Strode C, Wondji CS, David JP, Hawkes NJ, Lumjuan N, et al (2008) Genomic analysis of detoxification genes in the mosquito *Aedes aegypti* . Insect Biochem Mol Biol 38: 113–123. 18070670 10.1016/j.ibmb.2007.09.007

[34] Chandor-Proust A, Bibby J, Regent-Kloeckner M, Roux J, Guittard-Crilat E, et al (2013) The central role of mosquito cytochrome P450 CYP6Zs in insecticide detoxification revealed by functional expression and structural modelling. Biochem J 455: 75–85. 23844938 10.1042/BJ20130577 PMC3778711

[35] Stevenson BJ, Bibby J, Pignatelli P, Muangnoicharoen S, O'Neill PM, et al (2011) Cytochrome P450 6M2 from the malaria vector *Anopheles gambiae* metabolizes pyrethroids: Sequential metabolism of deltamethrin revealed. Insect Biochem Mol Biol 41: 492–502. 21324359 10.1016/j.ibmb.2011.02.003

[36] Stevenson BJ, Pignatelli P, Nikou D, Paine MJ (2012) Pinpointing P450s associated with pyrethroid metabolism in the dengue vector, *Aedes aegypti*: developing new tools to combat insecticide resistance. PLoS Negl Trop Dis 6: e1595. 22479665 10.1371/journal.pntd.0001595 PMC3313934

[37] Ding Y, Hawkes N, Meredith J, Eggleston P, Hemingway J, et al (2005) Characterization of the promoters of Epsilon glutathione transferases in the mosquito *Anopheles gambiae* and their response to oxidative stress. Biochemical Journal 387: 879–888. 15631620 10.1042/BJ20041850 PMC1135021

[38] Riveron JM, Yunta C, Ibrahim SS, Djouaka R, Irving H, et al (2014) A single mutation in the GSTe2 gene allows tracking of metabolically-based insecticide resistance in a major malaria vector. Genome Biol 15: R27. 24565444 10.1186/gb-2014-15-2-r27 PMC4054843

[39] Montella IR, Schama R, Valle D (2012) The classification of esterases: an important gene family involved in insecticide resistance - A review. Memórias do Instituto Oswaldo Cruz 107: 437–449. 22666852 10.1590/s0074-02762012000400001

[40] Silva AX, Jander G, Samaniego H, Ramsey JS, Figueroa CC (2012) Insecticide resistance mechanisms in the green peach aphid *Myzus persicae* (Hemiptera: Aphididae) I: A transcriptomic survey. PLoS One 7: e36366. 22685538 10.1371/journal.pone.0036366 PMC3369866

[41] Saavedra-Rodriguez K, Suarez AF, Salas IF, Strode C, Ranson H, et al (2012) Transcription of detoxification genes after permethrin selection in the mosquito *Aedes aegypti* . Insect Mol Biol 21: 61–77. 22032702 10.1111/j.1365-2583.2011.01113.x PMC3540788

[42] Grisales N, Poupardin R, Gomez S, Fonseca-Gonzalez I, Ranson H, et al (2013) Temephos resistance in *Aedes aegypti* in Colombia compromises dengue vector control. PLoS Negl Trop Dis 7: e2438. 24069492 10.1371/journal.pntd.0002438 PMC3777894

[43] David JP, Faucon F, Chandor-Proust A, Poupardin R, Riaz MA, et al (2014) Comparative analysis of response to selection with three insecticides in the dengue mosquito *Aedes aegypti* using mRNA sequencing. BMC Genomics 15: 174. 24593293 10.1186/1471-2164-15-174 PMC4029067

[44] Strode C, de Melo-Santos M, Magalhaes T, Araujo A, Ayres C (2012) Expression profile of genes during resistance reversal in a temephos selected strain of the dengue vector, *Aedes aegypti* . PLoS One 7: e39439. 22870187 10.1371/journal.pone.0039439 PMC3411583

[45] Ismail HM, O'Neill PM, Hong DW, Finn RD, Henderson CJ, et al (2013) Pyrethroid activity-based probes for profiling cytochrome P450 activities associated with insecticide interactions. Proc Natl Acad Sci U S A 110: 19766–19771. 24248381 10.1073/pnas.1320185110 PMC3856776

[46] Fry JD, Saweikis M (2006) Aldehyde dehydrogenase is essential for both adult and larval ethanol resistance in *Drosophila melanogaster* . Genet Res 87: 87–92. 16566845 10.1017/S0016672306008032

[47] Fry JD, Donlon K, Saweikis M (2008) A worldwide polymorphism in aldehyde dehydrogenase in *Drosophila melanogaster*: evidence for selection mediated by dietary ethanol. Evolution 62: 66–75. 18070084 10.1111/j.1558-5646.2007.00288.x

[48] Hemingway J, Hawkes NJ, McCarroll L, Ranson H (2004) The molecular basis of insecticide resistance in mosquitoes. Insect Biochem Mol Biol 34: 653–665. 15242706 10.1016/j.ibmb.2004.03.018

