# Supplementary material for: Identification and Characterisation of Aedes aegypti Aldehyde Dehydrogenases Involved in Pyrethroid Metabolism
Source: PLoS One. 2014 Jul 21;9(7):e102746. doi: 10.1371/journal.pone.0102746 (PMC4105619; doi:10.1371/journal.pone.0102746)
Supplement: Figure S1 — Phylogenetic relationship of ALDHs in Ae. aegypti (AAEL) with An. gambiae ALDHs (AGAP). (DOCX) [file pone.0102746.s001.docx]

**Figure S1**


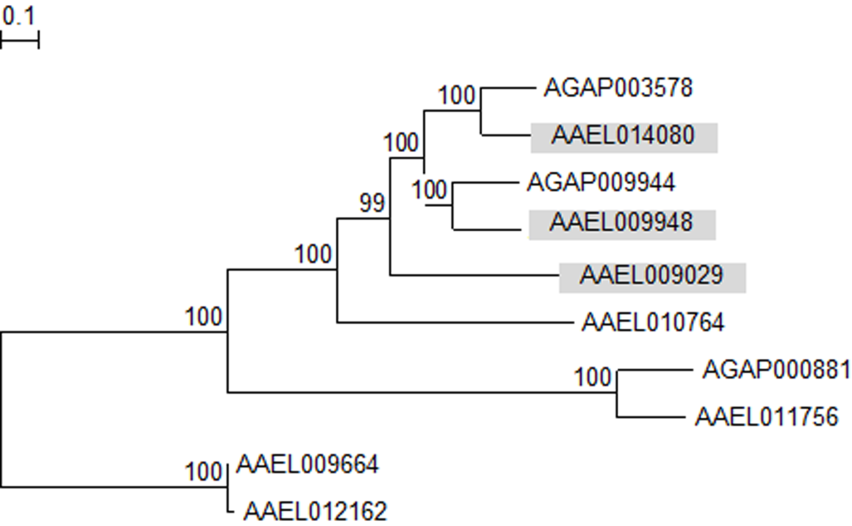


**Figure S1.** Phylogenetic relationship of ALDHs in *Ae. Aegypti* (AAEL) with *An. gambiae* ALDHs (AGAP). Amino acid sequences were aligned using ClustalW and a distance neighbor-joining tree was generated using TREECON (Van de Peer and De Wachter, 1993). Nodes with distance bootstrap values (1000 replicates) of 70% are shown. Three ALDHs used in this study are highlighted in gray.
