# Supplementary material for: Identification and Characterisation of Aedes aegypti Aldehyde Dehydrogenases Involved in Pyrethroid Metabolism
Source: PLoS One. 2014 Jul 21;9(7):e102746. doi: 10.1371/journal.pone.0102746 (PMC4105619; doi:10.1371/journal.pone.0102746)
Supplement: Figure S2 — SDS-PAGE analysis of His-tagged recombinant ALDHs in Ae. aegypti produced in E. coli BL21 Star (DE3). (DOCX) [file pone.0102746.s002.docx]

Figure S2


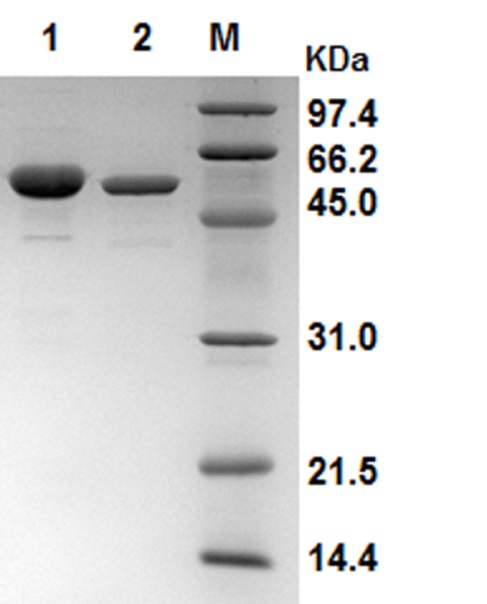


**Figure S2** SDS-PAGE analysis of His-tagged recombinant ALDHs in *Ae. aegypti* produced in *Escherichia coli* BL21 Star (DE3). Proteins were affinity purified and verified by 12.5% SDS-PAGE. Lane 1 and 2 represent recombinant ALDH 9948 and ALDH 14080, respectively. Lane M indicates the relative molecular masses of standard protein.
