# Supplementary material for: Identification and Characterisation of Aedes aegypti Aldehyde Dehydrogenases Involved in Pyrethroid Metabolism
Source: PLoS One. 2014 Jul 21;9(7):e102746. doi: 10.1371/journal.pone.0102746 (PMC4105619; doi:10.1371/journal.pone.0102746)
Supplement: Figure S3 — Chromatograms of PBald oxidation to PBacid by recombinant aldehyde dehydrogenase. (DOCX) [file pone.0102746.s003.docx]

Figure S3


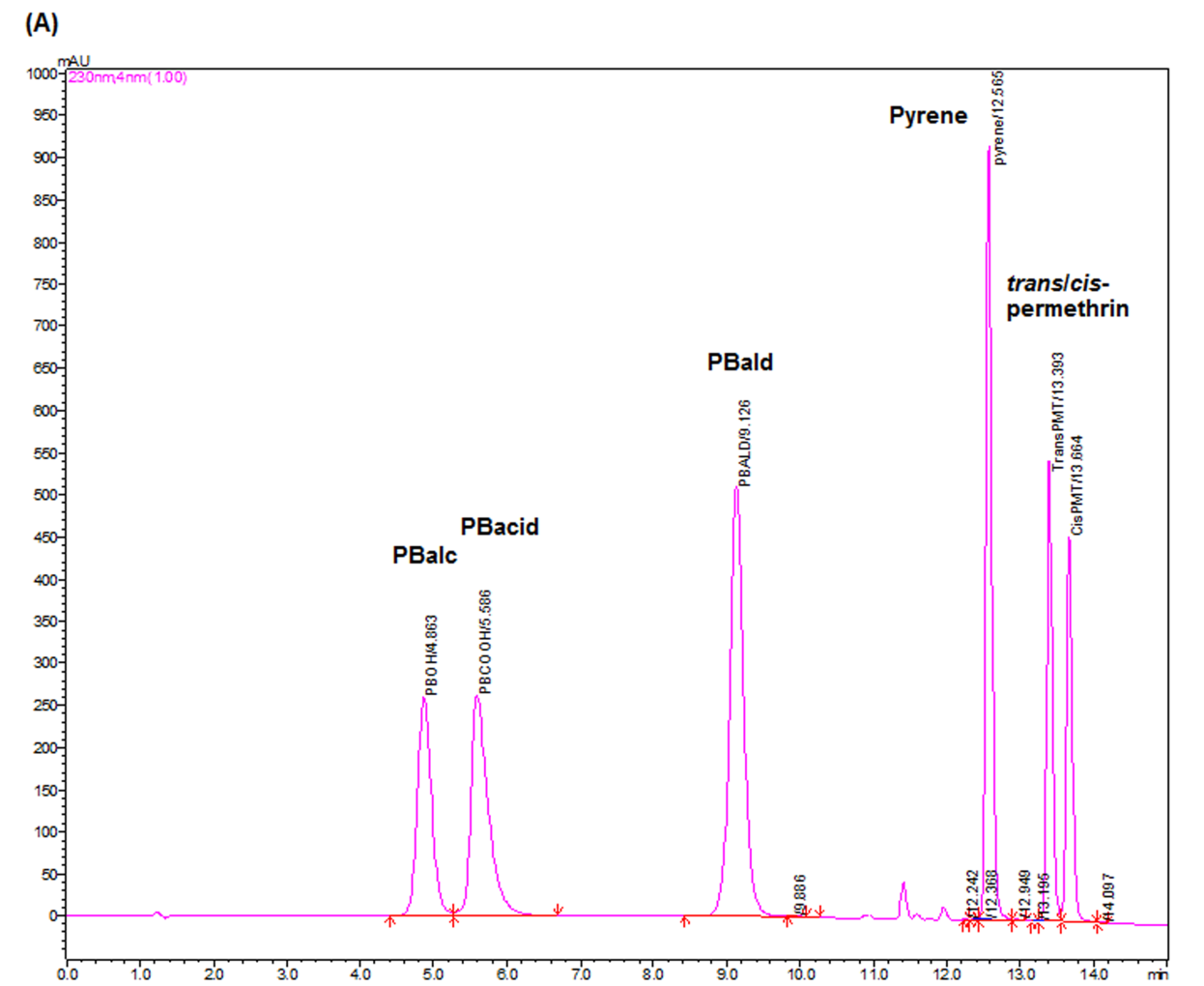


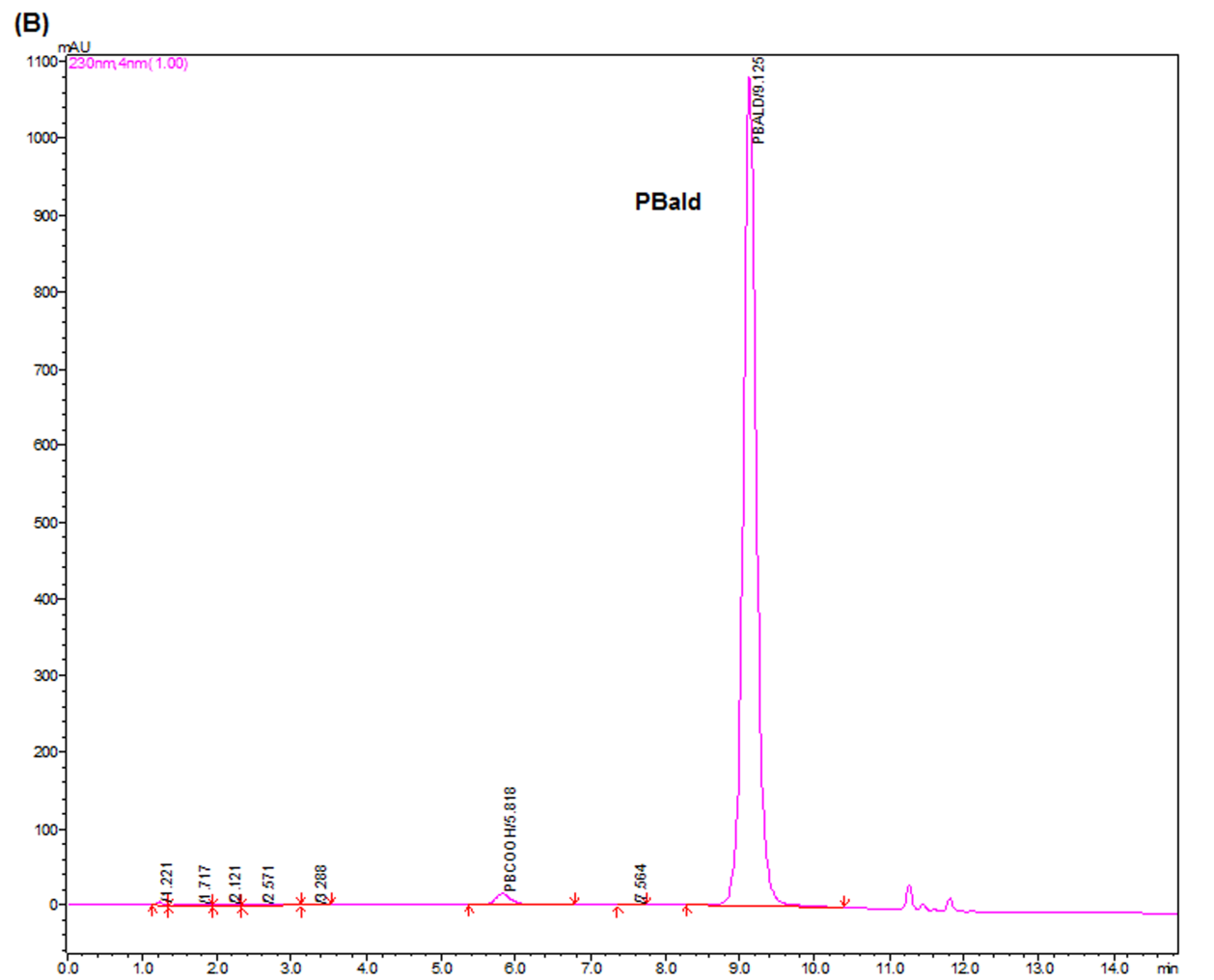


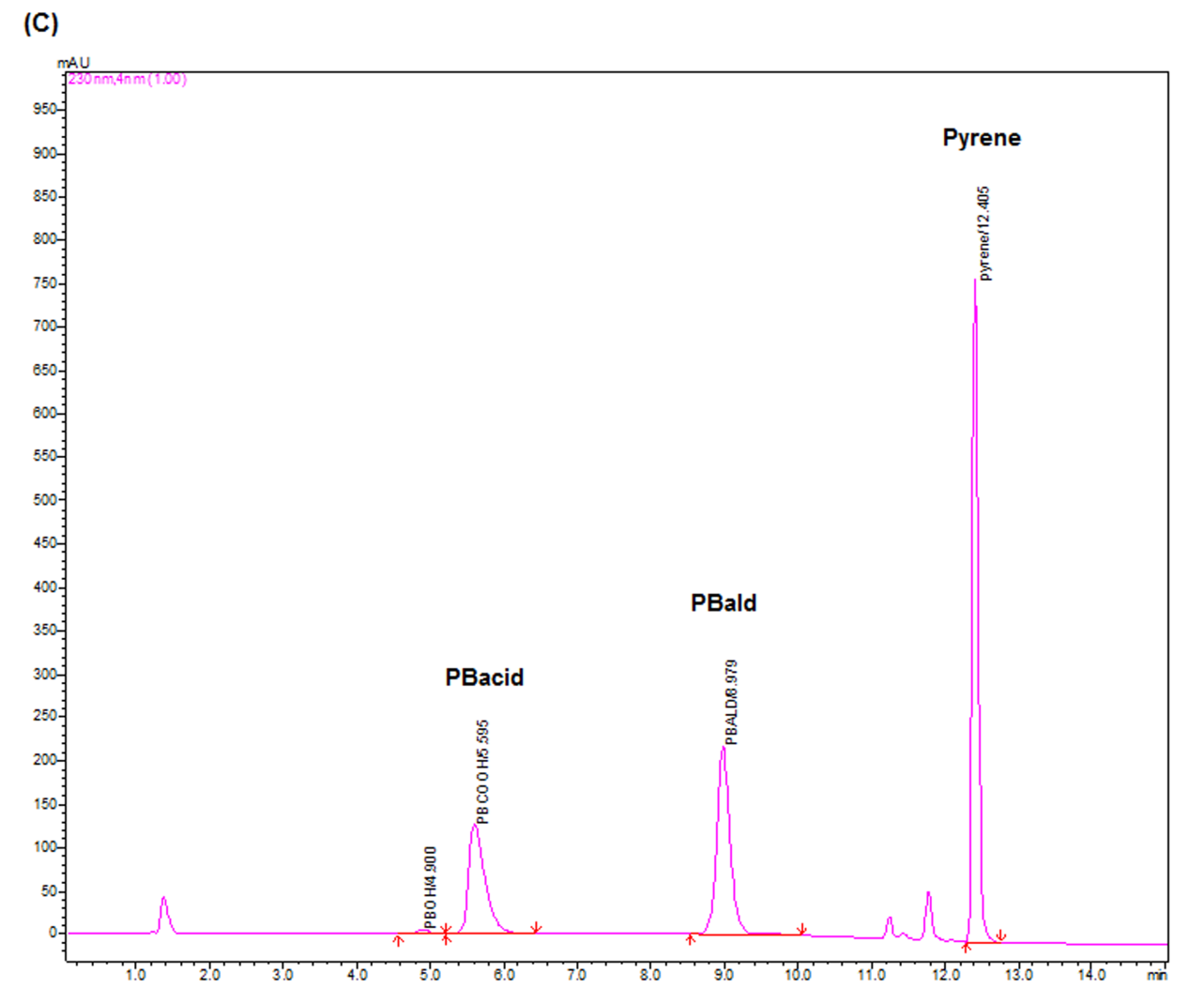


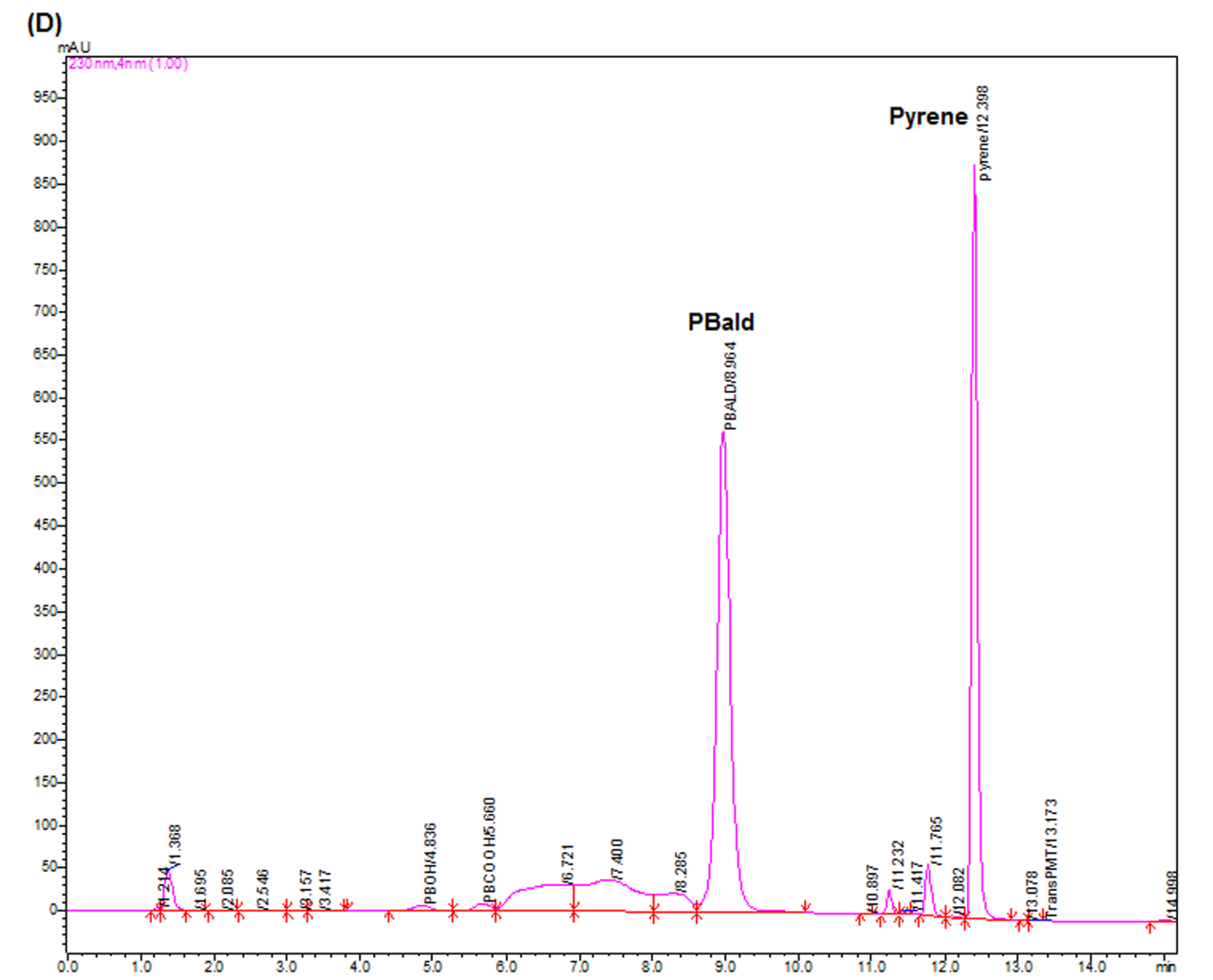


**FigureS3** Chromatograms of phenoxybenzylaldehyde (PBald) oxidation to phenoxybenzoic acid (PBacid) by recombinant aldehyde dehydrogenase. **(A)** Mixed standards containing 5 nmole of each standard per 10 µl injection of cis/trans-permethrin, phenoxybenzyl alcohol (PBalc), PBald and PBacid. Pyrene was spiked as an internal control. **(B)** Substrate PBald 10 nmole per 10 µl injection**(C)** Recombinant ALDH 14080 (20 µg) was incubated with 0.4 mM PBald in the presence of 3 mM NAD^+^ in 0.1 M Tris-Cl buffer pH 7.4 at 37ºC for 10 min. **(D)** Denatured recombinant ALDH 14080 was used as control. PBacid formation was determined by HPLC as described.
