# Supplementary material for: Identification and Characterisation of Aedes aegypti Aldehyde Dehydrogenases Involved in Pyrethroid Metabolism
Source: PLoS One. 2014 Jul 21;9(7):e102746. doi: 10.1371/journal.pone.0102746 (PMC4105619; doi:10.1371/journal.pone.0102746)
Supplement: Table S1 — Sequences of oligonucleotide primers used to amplify the cDNA full-length of ALDHs for in vitro protein expression. (DOCX) [file pone.0102746.s004.docx]

**Table S1.** Sequences of oligonucleotide primers used to amplify the full-length of *Ae. aegypti* ALDHs for *in vitro* protein expression.

| Gene | Primer name | Primer sequence (5’-3’) |
| --- | --- | --- |
| *ALDH9029* | ALDH9029F1 | CACCATGTTGCGCGTTTTG |
|  | ALDH9029R1 | TTATGAATTTTTGACTGGAATACG |
| *ALDH9948* | ALDH9948F1 | CACCATGGCTAACGCAAACC |
|  | ALDH9948R1 | CTAGACCTTAGACGGTAGCTTGATG |
| *ALDH14080* | AaALDHF | CACCATGGCCAATCCCAATC |
|  | AaALDHR | TCAGACCTTCGATGGCAGC |
