# Supplementary material for: Identification and Characterisation of Aedes aegypti Aldehyde Dehydrogenases Involved in Pyrethroid Metabolism
Source: PLoS One. 2014 Jul 21;9(7):e102746. doi: 10.1371/journal.pone.0102746 (PMC4105619; doi:10.1371/journal.pone.0102746)
Supplement: Table S2 — Sequences of oligonucleotide primers used to amplify the fragment of Ae. aegypti ALDHs for quantitative PCR. (DOCX) [file pone.0102746.s005.docx]

**Table S2**. Sequences of oligonucleotide primers used to amplify the fragment of *Ae. aegypti* ALDHs for quantitative PCR.

| Gene | Primer name | Primer sequence (5’-3’) |
| --- | --- | --- |
| *ALDH9029* | ALDH9029F | TCCCTATGGCCATCAACAAT |
|  | ALDH9029R | TTCATGACGGGTGTAAACGA |
| *ALDH9948* | ALDH9948F | GGTGGGAAAAATTGTGATGG |
|  | ALDH9948R | TCGTAGATTCCCTCCTGCAC |
| *ALDH14080* | ALDH14080F1 | GCTGGACAATGGCAAGGC |
|  | ALDH14080R | TTCCAGGGAATGATTTGACC |
| *SP7* | AaSP7F1 | gtacatcacccgcgctcgtg |
|  | AaSP7R1 | cttgtccaggtgcaccttg |
