# Supplementary material for: Identification and Characterisation of Aedes aegypti Aldehyde Dehydrogenases Involved in Pyrethroid Metabolism
Source: PLoS One. 2014 Jul 21;9(7):e102746. doi: 10.1371/journal.pone.0102746 (PMC4105619; doi:10.1371/journal.pone.0102746)
Supplement: Table S3 — Quantitative PCR results of Ae. aegypti ALDH. (DOCX) [file pone.0102746.s006.docx]

**Table S3**. Quantitative PCR results of *Ae. aegypti* *ALDH*

| Gene | Life stage | Ratio of copy number | | |
| --- | --- | --- | --- | --- |
|  |  | PMD/NO | PMDR/NO | PMDR/PMD |
| *ALDH9029* | Larva | 19.1^a^ | 3.7 | 0.2^a^ |
|  | Pupa | 23.2 | 24.5 | 1.1 |
|  | Male | 2.0 | 2.1 | 1.1 |
|  | Female | 2432.3^a^ | 170.4 | 0.1^a^ |
|  |  |  |  |  |
| *ALDH9948* | Larva | 0.6 | 1.8^b^ | 3.0^a^ |
|  | Pupa | 1.2 | 2.0^b^ | 1.7^c^ |
|  | Male | 0.7 | 0.9 | 1.3 |
|  | Female | 1.1 | 1.8^a^ | 1.6^a^ |
|  |  |  |  |  |
| *ALDH14080* | Larva | 0.4 ^c^ | 1.8^c^ | 4.0 ^a^ |
|  | Pupa | 1.1 | 1.2 | 1.0 |
|  | Male | 2.1^a^ | 1.5 | 0.7^c^ |
|  | Female | 1.5 | 1.6^c^ | 1.1 |

The gene transcript copy number was determined by normalising with the transcript copy number of ribosomal S7 transcript. The ratio of the average copy number was calculated by comparison with the average copy number of the New Orleans or PMD transcript. Statistically significant differences were evaluated with ANOVA followed by Tukey’s multiple comparison test (a, p<0.001; b, p<0.01; c, p<0.05 relative to New Orleans (NO) or PMD strain.
